# Investigation of the Evolutionary Patterns of Pore Structures and Mechanical Properties During the Hydration Process of Basalt-Fiber-Reinforced Concrete

**DOI:** 10.3390/ma18143212

**Published:** 2025-07-08

**Authors:** Junqin Zhao, Xuewei Wang, Fuheng Yan, Xin Cai, Shengcai Xiao, Shengai Cui, Ping Liu

**Affiliations:** 1 School of Civil Engineering, Sichuan Agricultural University, Chengdu 611830, China; junqin_zhao@163.com (J.Z.); 18208280744@163.com (F.Y.); 18116540663@163.com (X.C.); 18725644729@163.com (S.X.); 2 School of Civil Engineering, Southwest Jiaotong University, Chengdu 610031, China; 3 School of Civil Engineering, Chengdu University, Chengdu 610106, China; liupin@cdu.edu.cn

**Keywords:** basalt-fiber-reinforced concrete, watershed algorithm, mechanical behavior, pore variation, CT scan, multi-factor intensity mathematical model

## Abstract

Recent studies primarily focus on how the fiber content and curing age influence the pore structure and strength of concrete. However, The interfacial bonding mechanism in basalt-fiber-reinforced concrete hydration remains unclear. The lack of a long-term performance-prediction model and insufficient research on multi-field coupling effects form key knowledge gaps, hindering the systematic optimal design and wider engineering applications of such materials. By integrating X-ray computed tomography (CT) with the watershed algorithm, this study proposes an innovative gray scale threshold method for pore quantification, enabling a quantitative analysis of pore structure evolution and its correlation with mechanical properties in basalt-fiber-reinforced concrete (BFRC) and normal concrete (NC). The results show the following: (1) Mechanical Enhancement: the incorporation of 0.2% basalt fiber by volume demonstrates significant enhancement in the mechanical performance index. At 28 days, BFRC exhibits compressive and splitting tensile strengths of 50.78 MPa and 4.07 MPa, surpassing NC by 19.88% and 43.3%, respectively. The early strength reduction in BFRC (13.13 MPa vs. 22.81 MPa for NC at 3 days) is attributed to fiber-induced interference through physical obstruction of cement particle hydration pathways, which diminishes as hydration progresses. (2) Porosity Reduction: BFRC demonstrates a 64.83% lower porosity (5.13%) than NC (11.66%) at 28 days, with microscopic analysis revealing a 77.5% proportion of harmless pores (<1.104 × 10^7^ μm^3^) in BFRC versus 67.6% in NC, driven by densified interfacial transition zones (ITZs). (3) Predictive Modeling: a two dimensional strength-porosity model and a three-dimensional age-dependent model are developed. The proposed multi-factor model demonstrates exceptional predictive capability (R^2^ = 0.9994), establishing a quantitative relationship between pore micro structure and mechanical performance. The innovative pore extraction method and mathematical modeling approach offer valuable insights into the micro-structural evolution mechanism of fiber concrete.

## 1. Introduction

With the continuous progress of modern construction technology, the demands for the performance of building materials have become increasingly demanding. Although traditional concrete continues to be widely used, its inherent brittleness and susceptibility to cracking limit its applicability under complex stress conditions. To overcome this technical limitation, fiber reinforcement technology has been developed and applied. Among a wide variety of reinforcing materials, basalt fiber stands out due to its remarkable strength, corrosion resistance, and environmental friendliness, making it increasingly recognized as an ideal alternative to traditional synthetic fibers. As an emerging green inorganic fiber material, basalt fiber has seen widespread applications in concrete reinforcement in recent years, owing to its exceptional tensile strength, outstanding corrosion resistance, and eco-friendly properties [1,2]. However, the interfacial bonding mechanism governing the hydration reaction in basalt-fiber-reinforced concrete remains elusive, and the absence of an established long-term performance prediction model, coupled with insufficient research on multi-field coupling effects, constitute significant scientific knowledge gaps that hinder the systematic optimal design and broader engineering applications of basalt-fiber-reinforced concrete materials [3,4].

The incorporation of an appropriate amount of basalt fiber into concrete enables the preparation of basalt-fiber-reinforced concrete. Research has demonstrated that the addition of basalt fiber can significantly enhance the splitting tensile and flexural strengths of concrete [5,6,7,8]. Qin et al. [9] reported in their study that the workability of concrete decreases with the increasing mass and length of basalt fibers. Specifically, when the mass exceeds 3 kg/m^3^ and the length exceeds 24 mm, the rate of decline gradually slows down and eventually reaches a stable state. Y.G. Deng and Dias et al. [10,11] found that basalt fibers did not have a significant effect on the compressive strength of concrete when used alone and even reduced the mechanical properties with an increasing volume fraction. For example, when the volume fraction of basalt fibers was 1.0%, the compressive and splitting tensile strengths of concrete were 26.4% and 12% lower than those of ordinary concrete, respectively. Kizilkanat et al. [12] and Shen et al. [13] demonstrated that the incorporation of basalt fibers into concrete significantly enhances its tensile strength and improves toughness, deformation resistance, and the fracture modulus, thereby contributing to the overall durability and performance of the material. The hydration reaction, which acts as the primary internal influencing mechanism in concrete, plays a crucial role in determining its pore structure and mechanical properties. Specifically, this reaction directly controls the material’s pore distribution, the features of the interface transition zone, and its overall mechanical performance. After the incorporation of basalt fibers, their physical confinement effects and chemical interactions can significantly influence the hydration process of the cement matrix, thereby altering the development of the pore structure. Deng et al. [14] conducted a micro-structural analysis that demonstrated that the incorporation of basalt fibers promoted the formation of various fibrous and needle-like hydrated products within the composite material, effectively contributing to the filling or sealing of internal pores. Huang Daguan, C. Lian, et al. [15,16] established that the porosity of concrete was negatively correlated with both its compressive and flexural strengths. Basalt fiber can effectively reduce the total porosity of concrete, and an appropriate fiber volume fraction can further optimize the pore structure. As the basalt fiber content increases, the proportion of harmful pores decreases while the proportion of harmless pores increases. Specifically, when the fiber content is in the range of 0.2% to 0.25%, the proportion of harmful pores in the concrete reaches its minimum, and the proportion of harmless pores achieves its maximum.

Pores are a critical component of the micro-structure and meso-structure of concrete, directly influencing both the mechanical properties and durability of concrete. Chen et al. [17] showed that the ratio of compressive strength to indirect tensile strength (including splitting tensile and flexural strength) in cement mortar is not constant but instead correlates with its porosity. Specifically, this ratio decreases as the porosity of the cement mortar increases. Therefore, exploring the relationship between the porosity and strength of basalt-fiber-reinforced concrete during the hydration process is of significant importance for optimizing the material design and enhancing engineering applications [18,19,20,21]. Currently, CT scanning technology is widely employed as a critical tool for investigating concrete porosity. Numerous studies have utilized CT scanning technology to obtain images of various cross-sections through X-ray tomography, which are subsequently used to generate three-dimensional models via computer reconstruction for the analysis of the porosity and pore structure. This method is advantageous due to its non-destructive nature and its ability to provide detailed three-dimensional pore structure information, allowing for an accurate characterization of the pore size, shape, distribution, and connectivity [22,23,24,25,26,27]. Tian et al. [28] utilized X-ray computed tomography to systematically investigate the evolution of internal damage in concrete specimens subjected to repeated freeze–thaw cycles. Li et al. [29] explored the feasibility of using X-ray computed tomography for scanning intact concrete samples. By incorporating an in situ CT loading system, they successfully captured the internal structural changes caused by stress application. Uniaxial compression in situ X-ray computed tomography was performed on concrete specimens, allowing for the collection of real-time CT images that clearly illustrated the meso-scopic damage evolution within the material. Furthermore, regarding the relationship between the concrete strength and void ratio, a substantial number of formulas and models have been established in the existing literature [30,31,32,33,34,35,36]. However, the existing models primarily focus on static porosity–strength relationships but fail to account for time-dependent effects, limiting their applicability in predicting long-term performance.

Cui et al. [37] developed a multi-factor strength mathematical model to simulate the influence of the fractal dimension and composite porosity on concrete in hot–dry environments. This model accurately quantifies the relationship between the concrete strength and pore structure parameters. Song et al. [38] developed an empirical equation for predicting the reduction in pore tensile strength. However, most of these models are developed using non-standardized concrete test specimens and inadequately address the influence of fiber–matrix synergy and time on the porosity–strength relationship. As a key indicator of concrete material performance, the compressive strength significantly impacts the design of engineering structures. Therefore, while effectively managing test costs, achieving precise and rapid prediction of the compressive strength of basalt-fiber-reinforced concrete through data-driven approaches holds substantial research value.

To sum up, at present, most studies have concentrated on the static effects of the fiber content and curing age on the pore structure and strength of concrete. However, there is a lack of systematic analysis on the coupling mechanism of the dynamic pore evolution and the strength development during the concrete curing process. Concerning the influence of basalt fibers on the porosity evolution and the development of mechanical properties in the early stage of the hydration reaction, no consensus has been reached in the academic circle. In response to the above problems, this research combines the CT scanning technology and the pore extraction theoretical method based on the watershed algorithm to systematically study the pore evolution laws of basalt-fiber-reinforced concrete and normal concrete during the curing process and analyze their influences on the concrete strength. The core value of this study is to provide a scientific basis for the precise design and application of BFRC in specific real construction projects by establishing a clear, quantitative relationship between the microscopic pore structure and the macroscopic mechanical properties of basalt-fiber-reinforced concrete during the evolution of the dynamic hydration process, for example, predicting the behavior of structures at different ages to guide construction decisions, promoting the safe and reliable application of BFRC in specific construction environments and critical structures.

## 2. Material and Experimental Method

### 2.1. Materials

**Figure 1:**
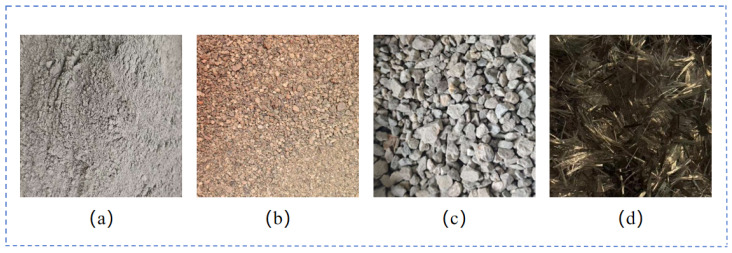
Raw Materials: (**a**) cement; (**b**) sand; (**c**) gravel; (**d**) basalt fiber.

The raw materials used in the experiment are illustrated in Figure 1, comprising water (density of 1000 kg/m^3^), cement (P·O·42.5, with a density ranging from 3100 to 3200 kg/m^3^, as depicted in Figure 1a), river sand (fineness modulus of 2.5, grading category II, as shown in Figure 1b), crushed stone (continuous grading of 5–10 mm, as presented in Figure 1c), and basalt fiber (density of 2650 kg/m^3^, single fiber diameter of 15 μm, length of 20 mm, as demonstrated in Figure 1d). This study involves normal concrete (named NC) and basalt-fiber-reinforced concrete (named BFRC). To minimize experimental error, three replicate samples were prepared for both NC and BFRC groups, with each test group containing three samples. For instance, the samples of ordinary concrete simultaneously included three different parallel test groups of NC1, NC2, and NC3, and the basalt fiber-reinforced concrete included three different parallel test groups of BFRC1, BFRC2, and BFRC3. The mechanical property test results were the average values obtained from the tests of the samples in the three parallel test groups. The raw material mix ratios of the NC and BFRC specimens are shown in Table 1.

### 2.2. Specimen Preparation

In this research, basalt-fiber-reinforced concrete with a fiber volume fraction of 0.2% was designated as the experimental group, while ordinary concrete served as the control group. For the pore structure evolution test conducted over the 28-day curing period, three cubic specimens (100 mm × 100 mm × 100 mm) were prepared for both the basalt-fiber-reinforced concrete and the normal concrete, resulting in a total of six specimens. Additionally, to evaluate the compressive strength and splitting tensile strength of the concrete at various ages (3, 5, 7, 9, 11, 14, 21, and 28 days), 48 cubic specimens (100 mm × 100 mm × 100 mm) were prepared for each type of concrete, yielding a total of 96 specimens. The specimen quantities are summarized in Table 2.

The preparation and curing process of concrete in this study referred to the JC/T 60018-2023 [39] officially released by China. The procedures for preparing the test specimens are described as follows: (1) accurately weigh the quantities of raw materials, including cement, sand, and crushed stones. Apply oil to the molds to facilitate easy release during demolding. (2) Moisten the mixer to verify its cleanliness and hydration status. Add cement and sand into the mixer and stir for 120 s until uniformly blended. Then, add crushed stones and continue stirring for an additional 120 s to produce dry-mixed materials. Subsequently, introduce water and a water-reducing agent solution in two stages. First, add part of the solution and stir for 60 s, followed by the remaining liquid while continuously stirring until complete homogeneity is achieved. (3) Once mixing is complete, fill the mold with concrete in layers, compacting it through vibration to eliminate air voids. (4) After leveling the surface, cover it with a protective film. (5) Let the molds and the specimens stand for 24 h until the initial setting, and then, carefully separate the specimens from the mold. (6) Finally, transfer the specimen to a constant-temperature water curing environment at 20 °C to ensure optimal strength development.

**Figure 2:**
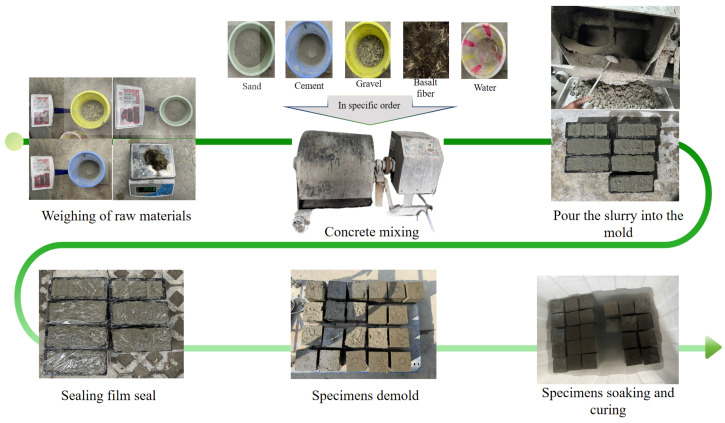
Flow chart of the specimen preparation.

During the fabrication of basalt-fiber-reinforced concrete specimens, the “agglomeration” phenomenon of basalt fibers in water presents a significant challenge for achieving uniform dispersion. To avoid non-uniform fiber distribution during mixing, which may impair the mechanical properties and durability of the concrete, the fibers are added in three separate batches after the introduction of crushed stones and are thoroughly mixed to ensure homogeneity. The experimental procedure is depicted in Figure 2.

### 2.3. Experimental Scheme

#### 2.3.1. Mechanical Strength Test

In accordance with the GB-50010-2010 [40] requirements, the specimens were subjected to compressive testing under specified loading conditions to determine the maximum load that they could withstand. Subsequently, compressive strength values were determined using Equation (1).

(1) $$f_{c}=\beta_{c}\frac{F_{c}}{A_{c}}$$

where

$f_{c}$ is the compressive strength (Mpa);
$F_{c}$ is maximum load at destruction of the test piece (kN);
$A_{c}$ is pressurized area of the specimen in mm^2^;
$\beta_{c}$ is the compression reduction factor, 0.95 for a specimen with a side length of 100 mm.

According to the GB/T-50081-2019 [41] requirements, the splitting tensile test loading was conducted. This method entails applying a compressive force perpendicular to the specimen’s axis, causing splitting failure and allowing for the indirect determination of its tensile strength. By recording the maximum load at the point of rupture, the tensile strength of the specimen can be calculated. The splitting tensile strength is represented by Equation (2).

(2) $$f_{st}=\beta_{st}\frac{2F_{st}}{\pi A_{st}}=0.637\beta_{st}\frac{F_{st}}{A_{st}}$$

where

$f_{st}$ is the splitting tensile of concrete (MPa);
$A_{st}$ is the splitting-surface area of specimens (mm^2^);
$\beta_{st}$ is the splitting tensile strength reduction factor, which is 0.85 for a specimen with the side length of a 100 mm reduction factor, which is 0.85 for a specimen with the side length of 100 mm.

**Figure 3:**
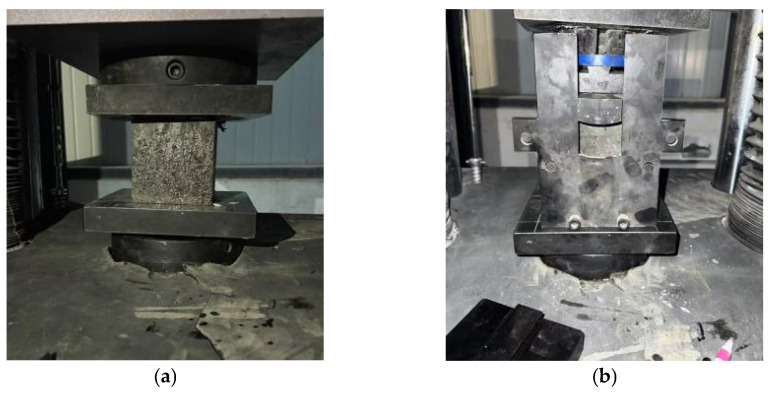

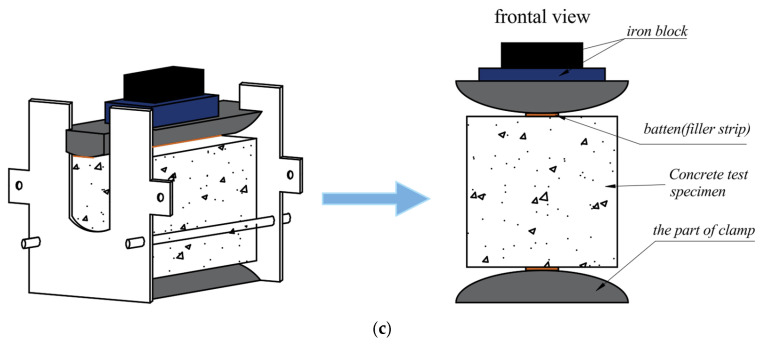
Mechanical testing machines for testing: (**a**) pressure resistance test machine; (**b**) split-resistant tensile test machine; (**c**) detailed schematic diagram of splitting tensile strength.

The experimental setup and testing principles employed in this study are illustrated in Figure 3. The experimental setup and testing principles includes a compression test machine (Figure 3a), a splitting tensile test machine (Figure 3b), and a schematic of the splitting test (Figure 3c).

#### 2.3.2. CT Scan

**Figure 4:**
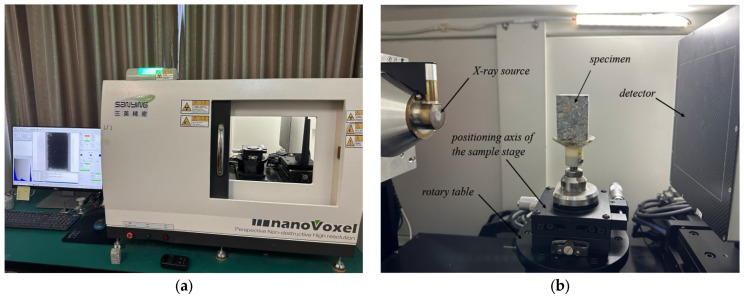

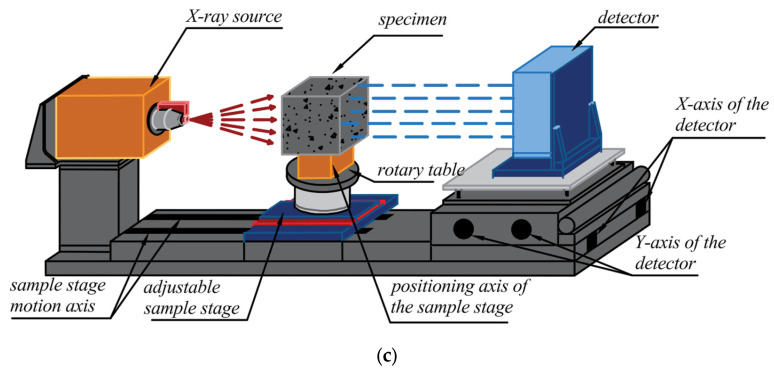
CT scanning equipment and procedure: (**a**) CT scanning equipment; (**b**) detailed CT scan image; (**c**) schematic diagram of CT scan.

CT scans of basalt-fiber-reinforced concrete were conducted daily over a 28-day period to collect data on the internal porosity, pore quantity, maximum pore diameter, and minimum pore diameter. It established a data basis for the quantitative analysis CT scanning technology, which can non-destructively obtain the internal pore and fiber distribution of concrete through X-ray tomography [22,23]. In this study, the nanoVoxel-1900 desktop micro-structure three-dimensional scanning imaging system (a CT scanning device) was employed to examine the pore evolution during the hydration process of basalt-fiber-reinforced concrete. Thereafter, the correlation between pore changes and mechanical strength was analyzed for the pore structure and mechanical properties. In this research, the NanoVoxel-1900 compact desktop CT system manufactured by Sanying Precision Instruments Co., Ltd., Tianjin Province, China. was employed for scanning experiments. Prior to scanning, 30 mm × 30 mm × 100 mm prismatic specimens were drilled from standard cubic specimens to serve as CT scanning samples to ensure the acquisition of representative internal structure information. Through several pre-tests, it was determined that the CT detection system voltage would be set at 100 kV, the current at 72 µA, the source to object distance (SOD) at 207 mm, and the source to detector distance (SDD) at 300 mm for the formal tests. During the scanning process, the platform was rotated 360 degrees with a final resolution of 1280 × 1280 pixels and a voxel size of 69.1 µm. Each scan took approximately 50 min to complete. The principle and equipment of the CT scanning experiment are presented in Figure 4.

#### 2.3.3. General Experimental Scheme

**Figure 5:**
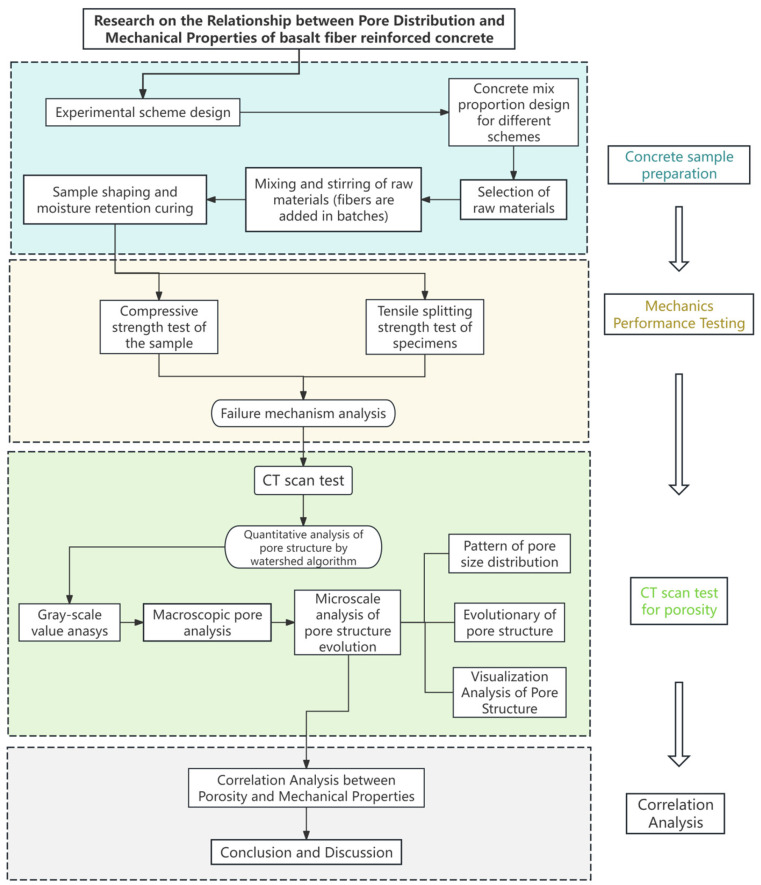
Research structure of the article.

Summarizing the experimental process discussed above, the general idea of the study in this paper is shown in Figure 5

## 3. Mechanical Properties

### 3.1. Failure Mechanism

**Figure 6:**
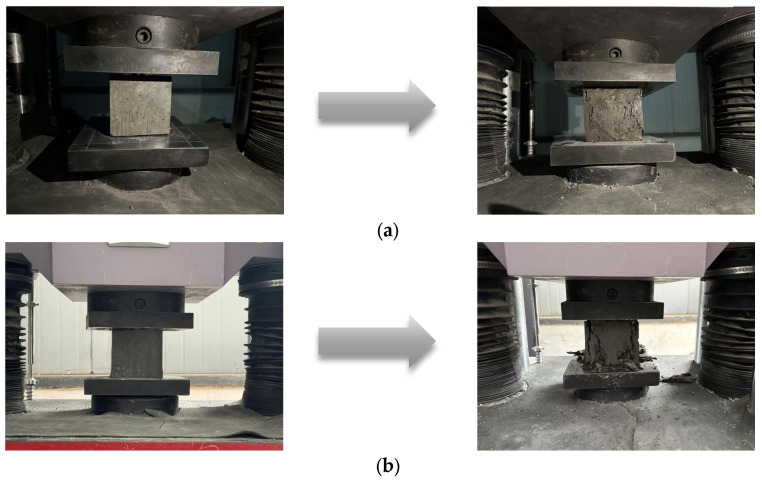
Schematic diagram of concrete destruction: (**a**) BFRC; (**b**) NC.

**Figure 7:**
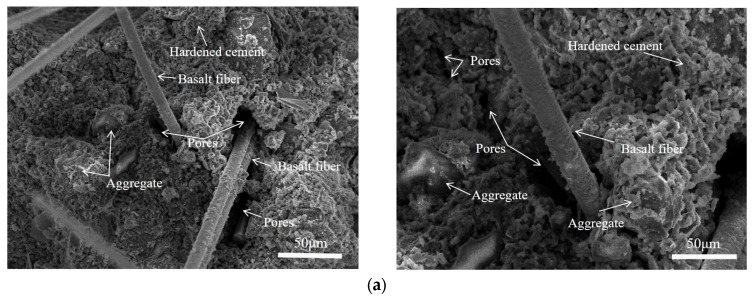

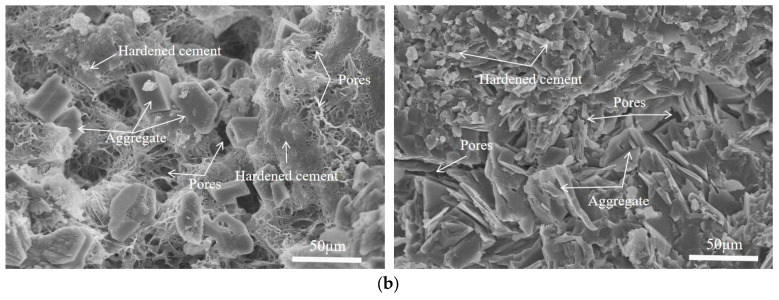
The SEM images of the sample: (**a**) BFRC; (**b**) NC.

The failure mode of concrete under compressive loading is illustrated in Figure 6. As shown in Figure 6a, basalt-fiber-reinforced concrete exhibits significant ductility at the point of failure. Conversely, Figure 6b reveals that ordinary concrete fails in a distinctly brittle manner. This difference in failure modes can primarily be attributed to the “bridging” effect of basalt fibers during crack propagation. By delaying the rapid penetration of cracks and altering the brittle failure mode of concrete through an energy dissipation mechanism, basalt fibers enhance the material’s performance. Moreover, the addition of basalt fibers facilitates a more uniform distribution of cement paste, thereby improving the bonding properties between cement particles to some extent and reducing shrinkage-induced cracks and pores. The specific effect is illustrated in Figure 7a, which displays the results of the SEM (scanning electron microscope) analysis. Figure 7b illustrates the SEM analysis results for normal concrete. The figures clearly reveal the surface morphology and structural characteristics of the sample under high magnification.

### 3.2. Mechanical Strength

**Figure 8:**
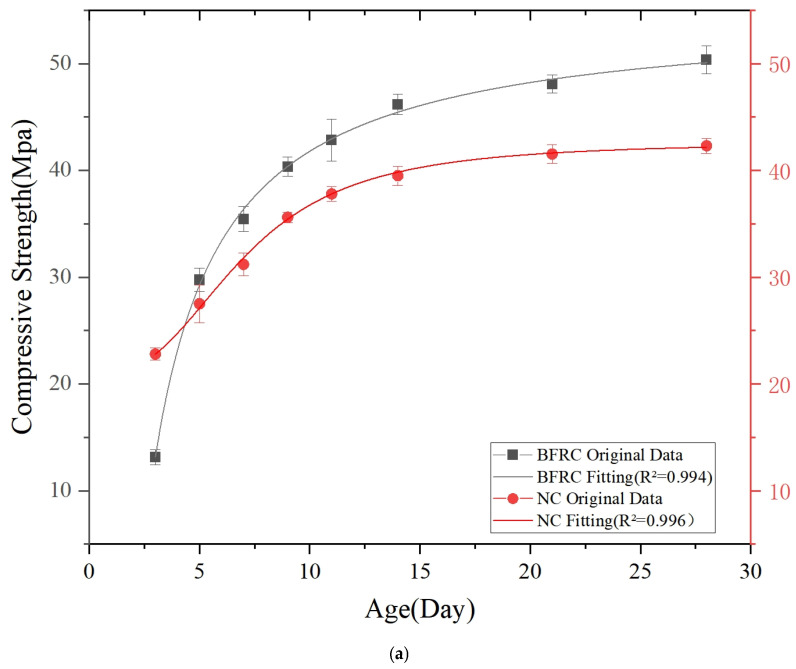

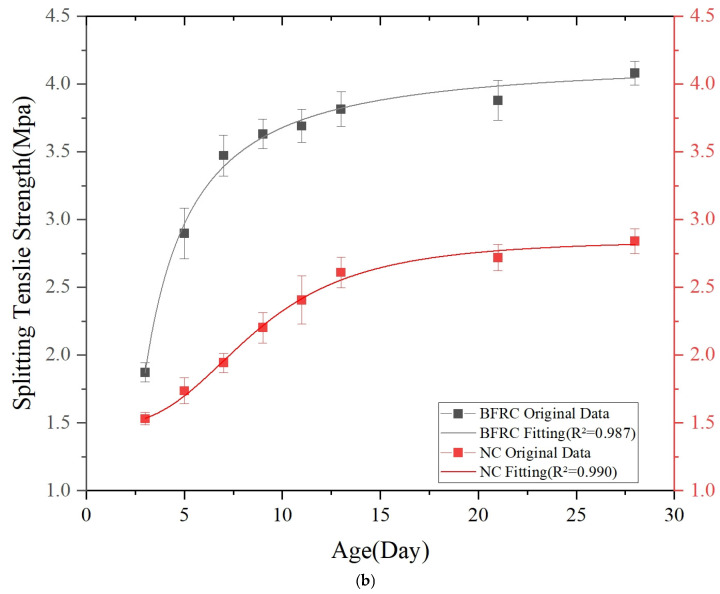
Variation in mechanical strength of concrete over a 28-day period: (**a**) compressive strength; (**b**) splitting tensile strength.

The compressive strength and splitting tensile strength tests for basalt-fiber-reinforced concrete and ordinary concrete were conducted at multiple time points, specifically at 3, 5, 7, 9, 11, 14, 21, and 28 days. Each group consisted of three cubic concrete specimens. According to the specification requirements, the strength of the cubic concrete specimens in each group was calculated as the average value of the experimental results from the three specimens. The compressive and splitting tensile strengths are summarized in Table 3 and Table 4, respectively. The mechanical strength test results of each group of samples are shown in Figure 8.

As illustrated in Figure 8a, the compressive strength of the BFRC group at 3 days was 13.13 MPa, which is significantly lower than that of the NC group at 22.81 MPa. This reduction can be attributed to the interference of fibers during the early stages of the cement hydration process. However, by day 5, the compressive strength of the BFRC group increased to 29.75 MPa, surpassing the NC group’s strength of 27.53 MPa and continuing to rise steadily thereafter. By day 28, the compressive strength of the BFRC group reached 50.78 MPa, indicating a 13.55% improvement over the NC group’s compressive strength of 42.36 MPa. The growth rate of the compressive strength of the BFRC group significantly accelerated after reaching 7 days of curing. This phenomenon can be attributed to the progressive densification of the interface between fibers and the matrix, thereby forming a three-dimensional reinforcing network structure. Such a structure not only enhances the load-transfer efficiency but also effectively restrains the propagation of internal defects.

As shown in Figure 8b, the splitting tensile strength of the BFRC group was consistently higher than that of the NC group across all ages. Specifically, at the age of 3 days, the splitting tensile strengths of the BFRC and NC groups were 1.87 MPa and 1.53 MPa, respectively, indicating a 22.2% increase in strength for the BFRC group compared to the NC group. At 28 days, the splitting tensile strength of the BFRC group reached 4.07 MPa, representing a 43.3% improvement over the NC group’s strength of 2.84 MPa. The growth rate of the splitting tensile strength in the BFRC group increases with age, exhibiting significant nonlinear growth after 7 days. This can be attributed to basalt fibers enhancing the interfacial bonding performance of cement-based composite materials and effectively improving the tensile properties of concrete [41].

## 4. Pore Analysis

### 4.1. Gray-Scale Value

The physical characterization of gray values in CT scan images and its application to identifying concrete components form a critical foundation for mesoscopic structure analysis in material science. Hong et al. [42] demonstrated that variations in the densities of different substances result in differences in X-ray attenuation coefficients, leading to distinct gray-scale representations of materials on scanned sections. Utilizing the principle of X-ray attenuation, CT technology generates grayscale images by recording the absorption differences of X-rays by various materials. In these images, the gray-scale value is positively correlated with the material density. For example, high-density coarse aggregates exhibit higher X-ray absorption and appear as high-gray-value regions (bright areas) in the image; low-density media such as air or water within pores correspond to low-gray-value regions (dark areas). Materials with intermediate densities, such as the cement slurry and fine aggregates, exhibit gray-scale characteristics that lie between those of high-density and low-density materials. Theoretically, threshold segmentation methods can be applied to distinguish internal pores, fine aggregates, and coarse aggregates within concrete. Cui et al. [43] conducted a study using CT scan images for segmentation and 3D reconstruction. In gray-scale imaging, an ellipsoid approximation method was applied to accurately characterize the morphology of individual pores, providing critical insights into the simulation of concrete pore structures.

**Figure 9:**
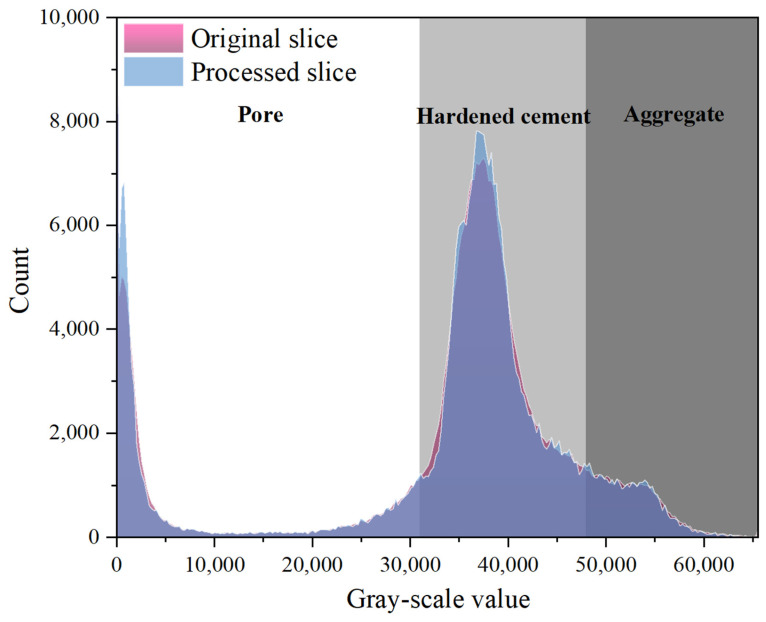
Gray-scale value image and classification.

The gray value directly quantifies the material density. Its quantification can be expressed in terms of intensity, and the intensity range maps the gray value to the distinguishable grayscale range of the human eye through the window width and window level. These parameters work together to improve the image contrast and diagnostic information. In this study, the gray value range was adjusted by modifying the intensity range, and three-phase segmentation of the concrete gray model was conducted using Avizo3D software 2022.2. The resulting three-phase gray thresholds for the concrete are presented in Figure 9.

Material porosity analysis based on CT imaging is a critical methodology for evaluating the characteristics of porous media in geotechnical materials. Currently, the calculation of porosity predominantly relies on the threshold segmentation method applied to CT images. However, this approach introduces uncertainties in defining the gray-level threshold that distinguishes pores from the solid matrix. Even when analyzing the same CT image, the use of different segmentation techniques can lead to significant variations in the calculated results. Feng et al. [44] utilized a CT image-processing algorithm to perform image segmentation and established a numerical model that accurately represents the micro-structure of EPS concrete specimens. Furthermore, Patra et al. [45] highlights that the traditional threshold segmentation method, which relies on engineers’ subjective judgment of gray values, faces significant challenges in accurately determining the threshold necessary for precisely segmenting the cement matrix from pores in in situ scanning images, especially when these images display subtle variations in gray values.

### 4.2. Watershed Algorithm

To address the aforementioned issues, this study proposes a porosity calculation method that integrates the gray-scale information of CT images with the watershed algorithm, thereby improving accuracy and reliability. This method integrates the concept of a digital ground elevation model and combines machine learning, gray recognition, and shape effects to determine a more accurate segmentation threshold. As a result, it facilitates the precise segmentation of pores and matrix in scanned specimens at various aging stages, thus providing a solid foundation for an additional investigation into pore evolution during the concrete hydration process.

**Figure 10:**
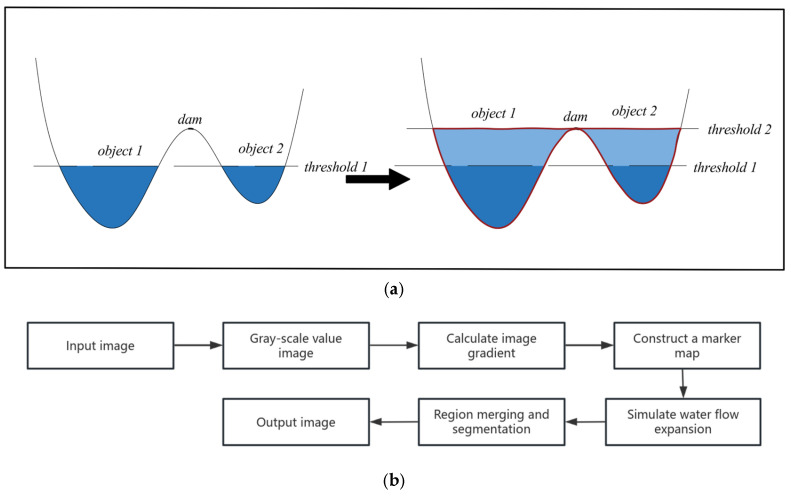
Watershed algorithm: (**a**) principle schematic representation; (**b**) operation flowchart.

The watershed algorithm is a segmentation technique that utilizes the topological structure of an image. By exploiting the gray-level information within the image, this method enables precise partitioning of the image into multiple distinct regions [46]. The watershed algorithm can be intuitively understood as modeling the image as a topographic surface, with high and low gray values corresponding to varying altitudes. By simulating the natural flow of water on this topographic surface, the algorithm performs efficient image segmentation. The fundamental principle and detailed procedural steps are depicted in Figure 10. Zhong et al. [47] employed a watershed algorithm (based on the multi-threshold H-maximum transform) combined with region merging correction, bilateral filtering, and Otsu-based coarse image segmentation techniques. This approach achieved particle size measurements with a standard deviation of less than 3% and demonstrated strong segmentation accuracy. These findings robustly confirm the feasibility and effectiveness of the watershed algorithm for micro-structure characterization.

### 4.3. Macroscopic Pore Analysis

#### 4.3.1. Pore Data Extraction Methodology

**Figure 11:**
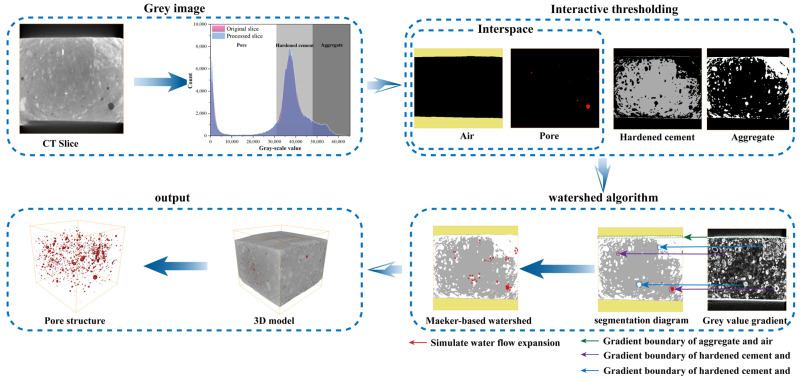
Flowchart for pore extraction using Avizo software.

To enhance the accuracy of porosity extraction for basalt-fiber-reinforced concrete specimens, an interactive threshold segmentation method was employed. This approach integrates Avizo3D software with the watershed algorithm to achieve more precise results. Firstly, the images of the specimens were scanned and subsequently processed in grayscale using Avizo3D software to generate the initial two-dimensional grayscale model (CT Slice) of the basalt-fiber-reinforced concrete specimens; secondly, interactive threshold processing is applied to the model. By employing a gray-level threshold, the two-dimensional gray-level model (CT Slice) is segmented into three distinct phases: interspace (including pore space and air), hardened cement paste, and aggregates; finally, a deliberate gap is retained at the gray value boundaries between each pair of adjacent phases. Subsequently, the watershed algorithm, which is based on the random forest model, is utilized for pore filling (simulating water flow expansion). This process facilitates the interconnection of connected domains within each pore, thereby ensuring the accurate segmentation of all three phases and ultimately generating a complete 3D model. The procedure for implementing grayscale modeling and pore data extraction is presented in Figure 11.

#### 4.3.2. Porosity Analysis

To investigate the development and evolution of pores during the hydration process of concrete, as well as the enhancement effect of basalt fibers on the pore structure, the porosity change rate, an index reflecting pore changes, was introduced, as shown in Equation (3), which enables a macroscopic analysis of pore evolution during the hydration process.

(3) $$\Delta P=\frac{\left(P_{1}-P_{2}\right)}{P_{1}}\times 100\%$$

where

ΔP is rate of change in porosity;
$P_{1}$ is porosity of concrete at period *t_1_*;
$P_{2}$ is porosity of concrete at period *t_2_*.

The variation in concrete porosity demonstrates significant nonlinear characteristics. Therefore, a logistic function fitting was conducted for the porosity of both basalt-fiber-reinforced concrete and ordinary concrete, with the resulting fitting equation presented in Equation (4).

(4) $$y=A_{2}+\frac{\left(A_{1}-A_{2}\right)}{\left[1+{\left(\frac{x}{x_{0}}\right)}^{p}\right]}$$

where

y is porosity (%);
x is time (days);
$A_{1}$ is the initial porosity (the y-value as x approaches 0);
$A_{2}$ is the final stable porosity (the y-value as x approaches infinity);
$x_{0}$ is a time-scale parameter that denotes the midpoint of the porosity change rate;
p is a shape parameter that quantifies the degree of steepness of the curve.

The parameters *A*_1_, *A*_2_, *x*_0_, and *p* in Equation (4) were estimated using the non-linear least squares method through the application of the Levenberg–Marquardt algorithm. The specific method is described as follows: firstly, the residual is defined, and its equation is provided in Equation (5). Secondly, the objective function is constructed by minimizing the sum of squared residuals, *S*, with its Equation presented in Equation (6). Finally, the fitting parameters for Equation (4) are determined, and the goodness of fit is quantified in Equation (7).

(5) $$r_{i}=y_{i}-{\hat{y}}_{i}=y_{i}-\left[A_{2}+\frac{\left(A_{1}-A_{2}\right)}{1+{\left(\frac{x_{i}}{x_{o}}\right)}^{p}}\right]$$

where

$r_{i}$ is the residual associated with the *i*-th observed value;
$y_{i}$ is the *i*-th observed value, and ${\hat{y}}_{i}$ represents the *i*-th predicted value

(6) $$S=\sum_{i=1}^{n}r_{i}^{2}=\sum_{i=1}^{n}{\left[y_{i}-\left(A_{2}+\frac{A_{1}-A_{2}}{1+{\left(\frac{x_{i}}{x_{0}}\right)}^{p}}\right)\right]}^{2}$$

where

S is the sum of the squared residuals.

(7) $$R^{2}=1-\frac{\sum r_{i}^{2}}{\sum {\left(y_{i}-\bar{y}\right)}^{2}}$$

where

$R^{2}$ is the coefficient of determination, indicating the goodness of fit.

**Figure 12:**
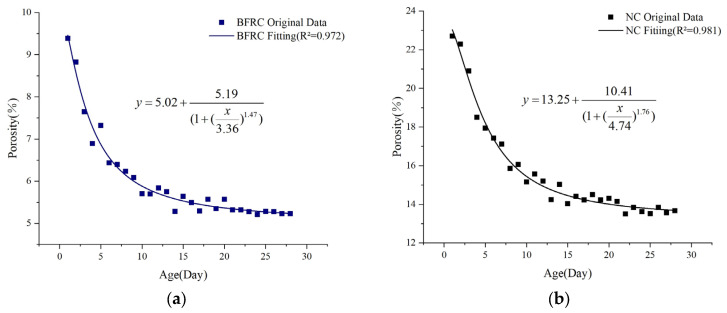
Regression analysis of concrete porosity variation: (**a**) BFRC; (**b**) NC.

**Figure 13:**
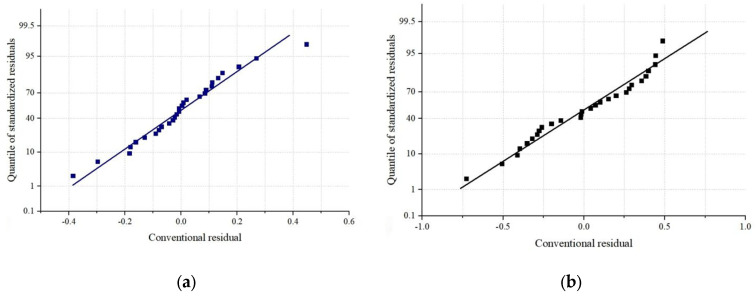
Variation of porosity difference in concrete: (**a**) BFRC; (**b**) NC.

The logistic function was utilized to model the shift in concrete porosity during the 28-day curing period. The regression curve results are displayed in Figure 12, and the residual distribution of the regression curve is shown in Figure 13.

It can be observed from Figure 12 and Equation (4) that **①** the weights assigned to *A*_1_ and *A*_2_ play a crucial role in determining the porosity range and constitute the core of the model’s output. For example, the significantly lower *A*_2_ value of BFRC indicates that the inhibitory effect of fibers on long-term porosity is predominant. **②** The weights assigned to *x_0_* and *p* play a critical role in governing the evolutionary process. For example, it can be observed from Figure 12a that the porosity of BFRC initially increases and subsequently decreases after 7 days. The inflection point time *x_0_* and the steepness *p* together reflect the fiber’s contribution to the later-stage strengthening effect.

It can also be observed from Figure 12 and Equation (4) that the porosity of basalt-fiber-reinforced concrete exhibits a decreasing trend over time. The rate of change gradually diminishes with time, eventually approaching stability. The porosity change rate of ordinary concrete is 39.3%, while that of basalt-fiber-reinforced concrete is 44.32%. When the water-to-binder ratio is fixed at 0.5 and the fiber volume fraction is uniformly distributed at 0.2%, the porosity of basalt-fiber-reinforced concrete decreases by 64.83% compared to that of ordinary concrete.

As shown in Figure 12, the fitting formulas for the porosity changes in basalt-fiber-reinforced concrete and ordinary concrete over time were established separately. The high R^2^ values of 0.972 and 0.981, respectively, indicate excellent fitting performance. Moreover, as depicted in Figure 13, the regression line of the standardized residual plot closely follows the y = x line, confirming that the residual distribution conforms to a normal distribution. This result also validates the reliability and robustness of the fitting results.

According to the pore theory, the porosity of concrete, as a porous material, plays a critical role in determining its compressive strength [37]. Tong et al. [48] demonstrated that a robust inter-facial bonding effect can be achieved between fibers and cement-based composite materials, thereby facilitating the formation of a spatial network structure within the specimens. This not only enhances the structural integrity but also effectively suppresses the initiation and propagation of pores. The inverse proportionality between porosity reduction and effective load-bearing area augmentation results in an improvement in compressive capacity per unit area. Consequently, it is anticipated that the compressive strength will increase as the porosity decreases.

### 4.4. Microscale Analysis of Pore Structure Evolution

#### 4.4.1. The Variation Pattern of Pore Size Distribution

**Figure 14:**
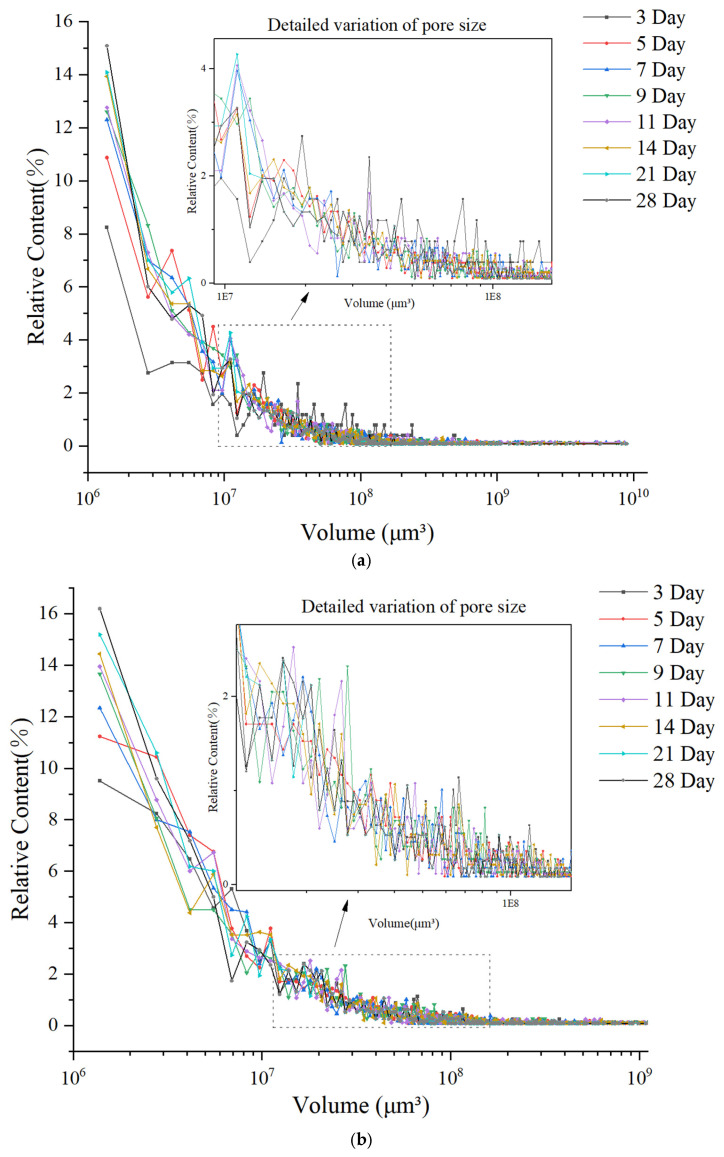

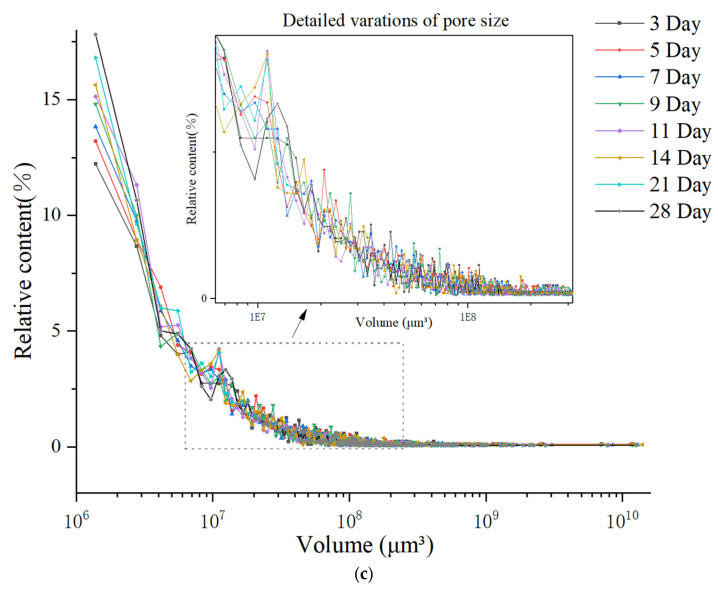
Microscopic pore size distribution in normal concrete: (**a**) NC1; (**b**) NC2; (**c**) NC3.

**Figure 15:**
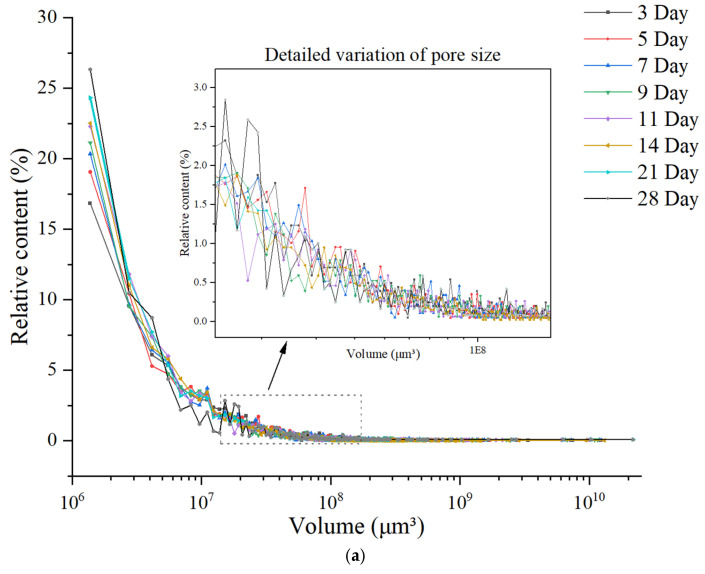

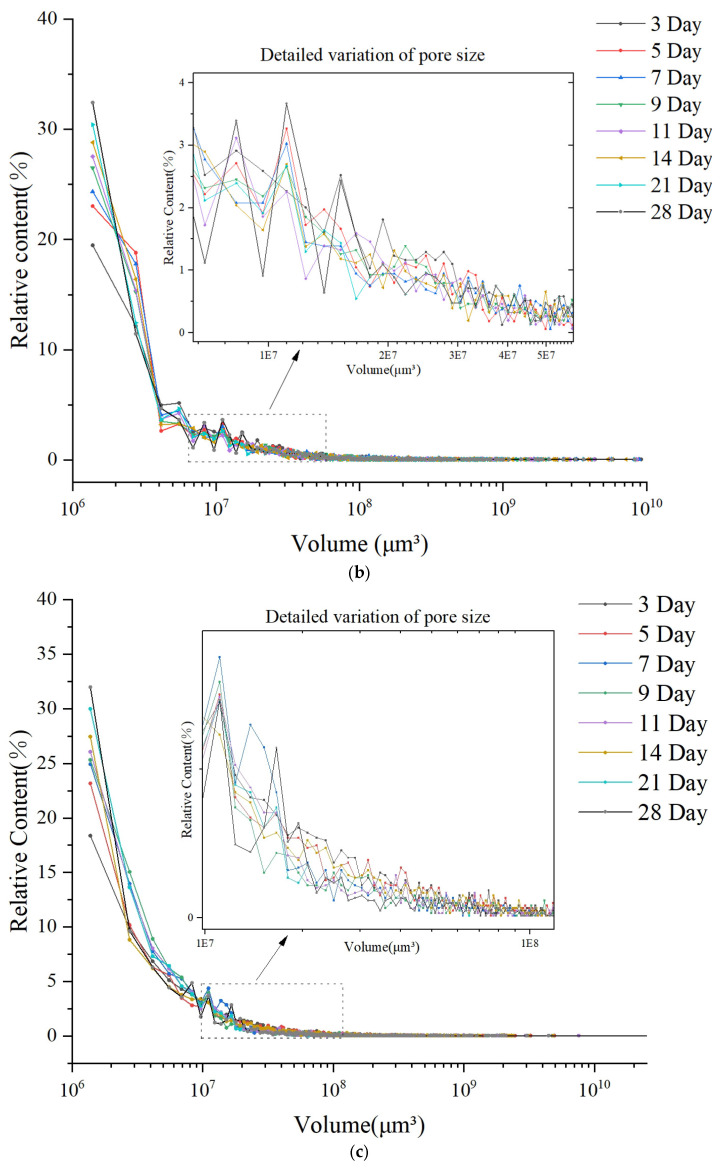
Microscopic pore size distribution in basalt-fiber-reinforced concrete: (**a**) BFRC1; (**b**) BFRC2; (**c**) BFRC3.

To investigate the variation law of pore size and the evolution process of pore structure during the hydration of concrete, this study systematically extracted and analyzed the pore parameters in concrete within the 28 days of curing. Figure 14 shows the relationship curve between pore size and its relative content in ordinary concrete from three groups of parallel tests, while Figure 15 depicts the corresponding curve for basalt-fiber-reinforced concrete from three groups of parallel tests.

As shown in Figure 14 and Figure 15, both basalt-fiber-reinforced concrete and ordinary concrete are predominantly composed of harmless pores with a volume of less than 1 × 10^7^ μm^3^. The proportion of these small voids increases gradually over time. At the age of 3 days, the relative content of small voids in basalt-fiber-reinforced concrete is higher than that in ordinary concrete. Furthermore, as the curing age increases, the growth rate of the relative content of small voids in fiber-reinforced concrete exceeds that observed in ordinary concrete. The subsequent text will conduct a detailed quantitative analysis of pore characteristics, providing insights into their specific properties and distributions.

#### 4.4.2. Analysis of the Evolutionary of Pore Structure

This research presents a threshold optimization method based on a statistical analysis. The proposed method aims to identify characteristic thresholds within the pore size distribution data of porous media, enabling a more systematic and in-depth exploration of the relative proportions of different pore sizes across the entire distribution. By analyzing the size distribution of all pores in the concrete during the 28-day curing period, a reasonable threshold was determined using a Python3.7.4-based algorithm for data segmentation. Subsequently, the pores were classified into two distinct categories: harmful pores and harmless pores, based on their specific sizes. The detailed procedure is presented as follows:

The initial step involves data preprocessing and import. Specifically, aggregate all pore data within the concrete into an Excel table and construct a two-dimensional data matrix. This matrix should consist of n observed samples and m pore types, as defined in Equation (8).

(8) $$D={\left[d_{ij}\right]}_{n\times m}(i=1,\ldots \ldots ,n;j=1,\ldots \ldots ,m)$$

where

D is the data matrix of aperture distribution, with dimensions n × m, where n is to the number of rows and m corresponds to the number of columns.
$d_{ij}$ is the matrix element corresponding to the measurement value of the aperture unknown in the j-th spatial dimension for the i-th sample.
n is the total number of independently observed samples.
m is the total number of unique pore types.

The second step involves constructing a relative content calculation model. The relative content index *RC_j_ (τ)* under the threshold *τ* is defined as the proportion of samples in the j-th column that meet the condition *d_i_*_j_ ≤ *τ*, as shown in Equation (9).

(9) $$RC_{j}\left(\tau \right)=\frac{1}{n}\sum_{i=1}^{n}I\left(d_{ij}\leq \tau \right)$$

where

$I\left(\cdot \right)$ is an indicator function, characterizing the cumulative probability distribution of aperture values that are less than or equal to the threshold τ.
$RC_{j}(\tau )$ is the cumulative frequency of the aperture at the J-column position where the aperture is less than or equal to *τ*.
τ is the aperture screening threshold.

The third step involves the application of a threshold optimization algorithm. The candidate threshold set {*τ_k_*} is constructed based on the unique value set of the first column of data, and the optimal threshold is determined via an iterative search process.

- For each *τ_k_*, calculate the m-dimensional relative content vector RC (*τ_k_*), represented as *τ_k_* = *RC (τ_k_) =* [*RC_1_ (τ_k_), …, RC_m_ (τ_k_)*].
- Assess the monotonicity conditions between adjacent positions as defined in Equation (10).

(10) $$\sum_{j=1}^{m-1}H(RC_{j+1}(\tau_{k})RC_{j}(\tau_{k}))\geq m$$

where

The Heaviside step function, denoted as H(x), is formally defined as follows: H(x) = 1 for *x* > 0, and H(x) = 0 for *x* ≤ 0.

c. Select the first *τ_k_* that fulfills the specified conditions as the optimal threshold τ*, ensuring that it aligns with the predefined criteria for selection.

The fourth step involves conducting visual verification. The value of *τ* obtained from the aforementioned algorithm is 1.104 × 10^7^ μm^3^. The histogram visualization of the relative content distribution corresponding to the optimal threshold τ is presented, with the horizontal axis denoting the spatial position index and the vertical axis indicating *RC_j_ (τ)*. The gradient characteristic of the pore size distribution is validated based on the columnar features.

**Figure 16:**
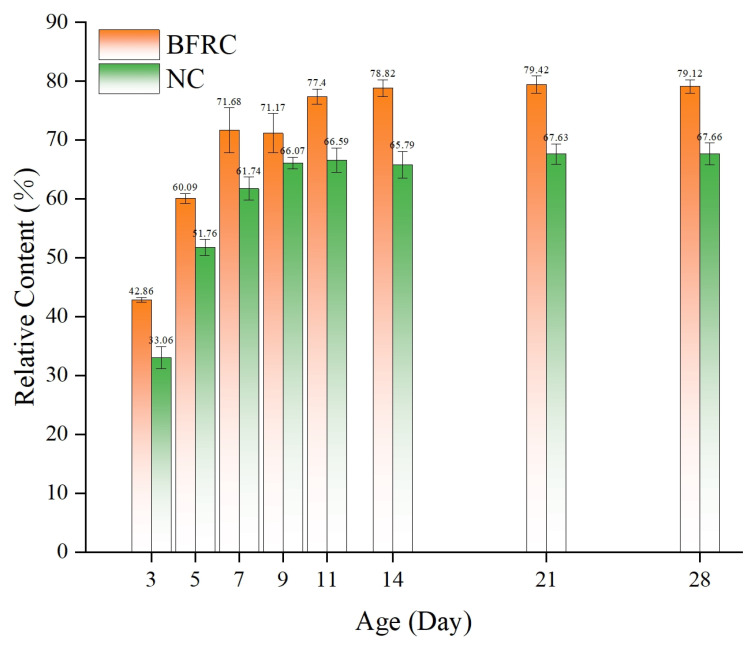
The relative proportion of harmless pores in concrete.

This algorithm conducted separate statistical analyses of the daily changes in the relative micro-pore content for both ordinary concrete and basalt-fiber-reinforced concrete over a 28-day curing period. Additionally, it investigated the hydration process of concrete at the microscopic level, providing a quantitative analysis of the complex chemical reactions between the major mineral components in Portland cement and water, as well as the enhancing effect of basalt fibers on this reaction. The data results are represented in Figure 16.

The relative proportion of harmless pores in ordinary concrete increased from 33.1% ±1.2% to 67.6% ±3.2%. With the incorporation of 0.2% basalt fiber, the relative proportion of tiny-pore structures in basalt-fiber-reinforced concrete rose from 42.8% to 77.5 ± 0.4%. Figure 15 demonstrates the daily variations in the relative proportions of micro-pores in both ordinary concrete and basalt-fiber-reinforced concrete over a 28-day period.

Consistent with previous reports [14], basalt fibers form a spatial network structure via a three-dimensional random distribution, effectively inhibiting the propagation of macroscopic defects. The chemically reactive surface of the fibers promotes the oriented growth of hydration products within the fiber–slurry inter-facial region, thereby enhancing the densification of nanoscale C-S-H gels. Li et al. [21] proposed that the gradient regulation mechanism of this pore structure can be attributed to the fiber–matrix interface effect, which critically enhances stress transfer and mechanical compatibility, thereby effectively regulating the structural performance.

Huang et al. [15] conducted a study on the non-destructive evaluation of the pore structure in basalt–polyvinyl alcohol hybrid fiber-reinforced concrete (BPHFRC) using nuclear magnetic resonance (NMR). Their findings revealed that during the hydration process of concrete containing 0.2% basalt fiber (BF), an optimal effect was observed: the incorporation of an appropriate amount of fibers promoted a more uniform three-dimensional distribution within the concrete matrix. This significantly improved the pore structure of the concrete and substantially reduced the proportion of multi-dimensional pores. Nevertheless, an excessive fiber content may result in fiber agglomeration, increased porosity, and compromised mechanical performance.

#### 4.4.3. Visualization Analysis of Pore Structure

**Figure 17:**
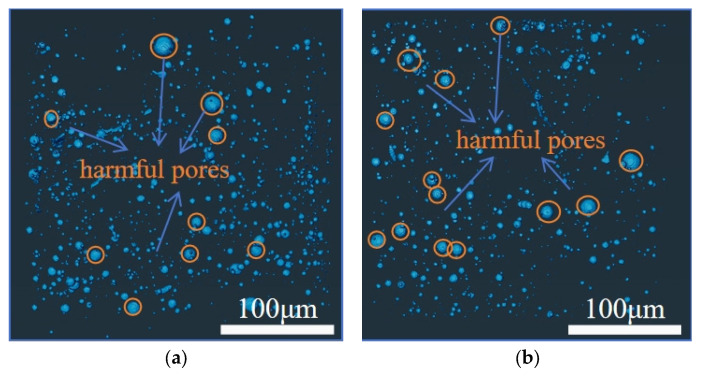

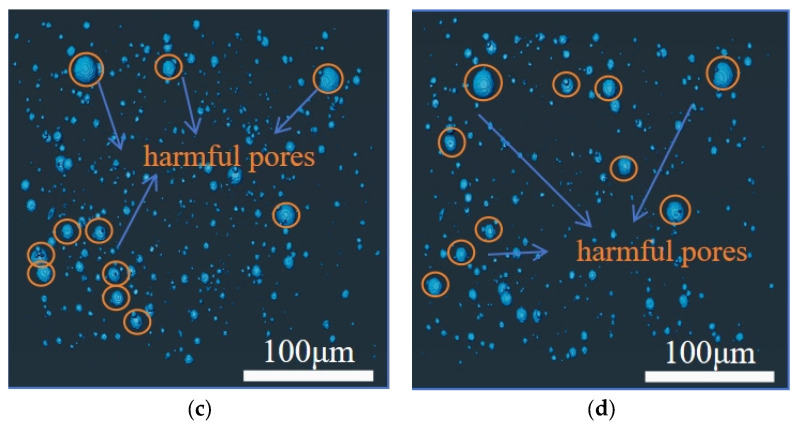
Schematic diagram of micro-structure: (**a**) NC-2 d; (**b**) NC-8 d; (**c**) NC-14 d; (**d**) NC-21 d.

**Figure 18:**
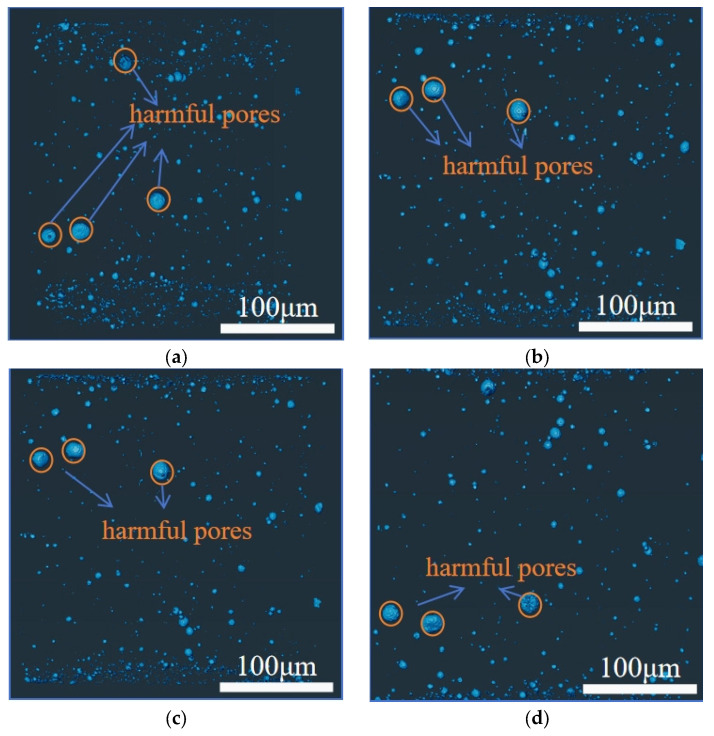
Schematic diagram of basalt fiber improved micro-structure: (**a**) BFRC-2 d; (**b**) BFRC-8 d; (**c**) BFRC-14 d; (**d**) BFRC-28 d.

To enhance the intuitive understanding of pore structure distribution, grayscale images of concrete pores are presented in Figure 17 and Figure 18. Figure 17 shows panels (a) to (d), which sequentially depict the pore conditions of ordinary concrete at the ages of 2 days, 8 days, 14 days, and 28 days. Similarly, Figure 18 illustrates panels (a) to (d), representing the pore characteristics of basalt-fiber-reinforced concrete at the corresponding ages.

By analyzing and comparing the variations in the relative micro-pore contents of ordinary concrete and basalt-fiber-reinforced concrete specimens within the same curing period, the optimization effect of basalt fiber on pore structure formation during the hydration process of concrete can be more clearly illustrated. In the cement paste, fibers are effectively integrated into the matrix, thereby limiting the expansion of capillary pores and improving the material’s overall performance. It can be observed that the incorporation of basalt fibers commonly reduces the porosity of concrete, thereby compacting its micro-structure and simultaneously decreasing the pore size. From the perspective of enhancing cement hydration, basalt fiber, as an inorganic material, may exhibit limited yet significant chemical interactions with specific cementitious components when incorporated into concrete. These interactions can facilitate the cement hydration process by reducing the number of unhydrated cement particles and minimizing the potential for pore formation. Moreover, the fibers can alter existing pore structures, enabling the cement mortar to infiltrate and fill these pores more effectively, thus optimizing the overall pore structure and improving the material’s mechanical properties.

Yang et al. [49] revealed from a microscopic perspective that the incorporation of fibers strengthens the effective inter-facial bonding between recycled aggregates and cement mortar, thereby promoting the formation of cohesive composites. This enhancement not only boosts the overall density of the sample but also refines the pore structure and suppresses the propagation of internal micro-cracks. As a result, the compressive strength and impermeability of recycled concrete are substantially enhanced.

## 5. Correlation Analysis Between Porosity and Mechanical Properties

**Figure 19:**
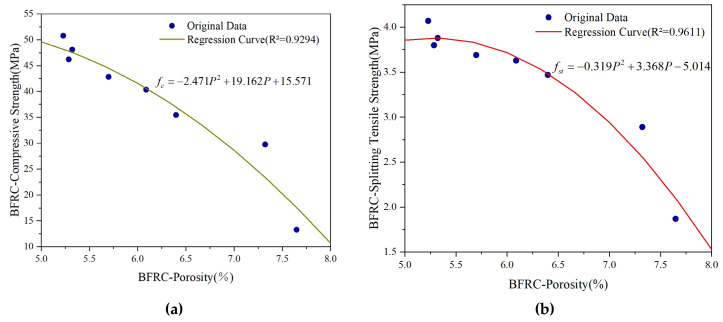
Regression curve of BFRC strength and composite porosity: (**a**) BFRC compressive strength and composite porosity; (**b**) BFRC splitting tensile strength and composite porosity.

**Figure 20:**
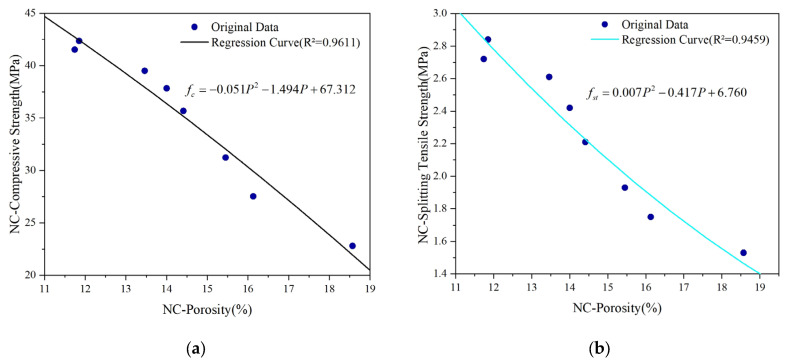
Regression curve of NC strength and composite porosity: (**a**) regression curve of NC compressive strength and composite porosity; (**b**) regression curve of tensile NC splitting tensile strength and composite porosity.

The strength of concrete, which serves as a fundamental indicator of its macroscopic properties, has been shown to be closely correlated with the pore structure. To quantitatively explore the relationship between strength and porosity, extensive research has been carried out in the academic community. For instance, Bian et al. [34] quantitatively evaluated the total influence coefficient of the pore size by integrating the pore size distribution function with the influence function. Subsequently, by incorporating this coefficient into the relationship between the effective strength and porosity, they successfully derived formulas for both the effective tensile and compressive strengths of porous concrete. Jin et al. [50] conducted a comprehensive analysis of the strength and pore structure of 13 types of Portland cement mortar mixtures, establishing a power function relationship between compressive strength and the ratio of the fractal dimension to capillary pore volume. Furthermore, a significant number of studies have systematically summarized diverse mechanical formulas and established corresponding mathematical models [32,33,34,35,36]. Through an analysis, it is evident that as time progresses, both basalt-fiber-reinforced concrete and ordinary concrete exhibit specific variation patterns in terms of the porosity and mechanical strength. Furthermore, their fitted curves reveal similar nonlinear variation trends. Based on this, a two-dimensional fitting model has been developed to quantitatively describe the relationship between porosity and strength. The resulting fitting curves are illustrated in Figure 19 and Figure 20, respectively.

As observed in Figure 19 and Figure 20, there is a strong fitting relationship between the porosity variation of concrete and its mechanical properties. The determination coefficients (R^2^) for the curve fitting are 0.9294, 0.9611, 0.9611, and 0.9456, respectively, indicating a high level of correlation.

To achieve a more precise mathematical model, the effect of curing age is incorporated into the analysis, and a multi-factor intensity prediction model is proposed, as shown in Equation (11).

(11) $$f=k_{0}+k_{1}P^{2}+k_{2}T^{2}+k_{3}PT+k_{4}P+k_{5}T$$

where

f is the strength of the concrete (MPa);
$k_{i}\;(i=1-5)$ is the regression coefficient;
$k_{0}$ is the intercept coefficient;
T is the time (days);
P is the porosity (%).

Based on Equation (11), the interaction terms of time and porosity are integrated into the model. Thereafter, the relationships among concrete strength, porosity, and other parameters are established through multiple regression analysis. Specifically, the strength equations for the multi-factor strength model of basalt-fiber-reinforced concrete are given by Equations (12) and (13), respectively. In contrast, the strength equations for the multi-factor strength model of ordinary concrete are presented by Equations (14) and (15), respectively.

(12) $$f_{c}=16.7478P^{2}+0.0749T^{2}-251.689P-31.0429T+5.4055PT+928.0349$$

(13) $$f_{st}=0.3839P^{2}+0.0036T^{2}-6.6254P-1.3508T+0.2354PT+28.7824$$

(14) $$f_{c}=9.8077P^{2}+0.0043T^{2}-161.2395P-23.569T+3.9746PT+647.8947$$

(15) $$f_{st}=0.0489P^{2}+0.0004T^{2}-1.7542P-0.4017T+0.0335PT+16.5944$$

where

$f_{c}$ is the compressive strength of concrete, MPa;
$f_{st}$ is the splitting tensile strength of concrete, MPa.

**Figure 21:**
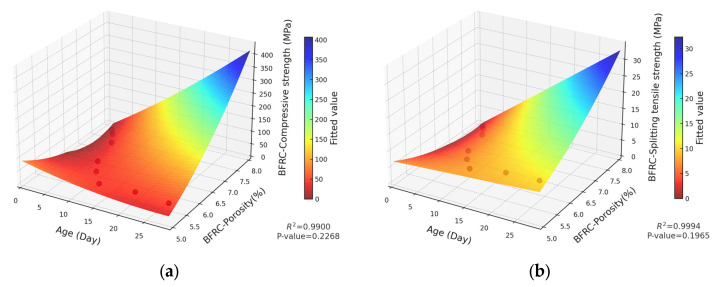
(**a**) Multiple regression of BFRC compressive strength; (**b**) multiple regression of BFRC splitting tensile strength.

**Figure 22:**
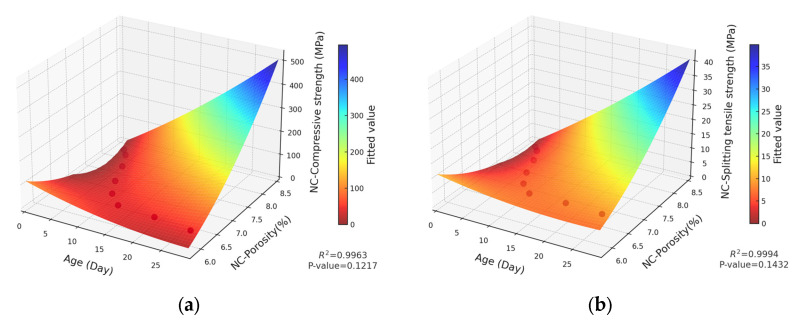
(**a**) Multiple regression of NC-compressive strength; (**b**) multiple regression of NC-tensile splitting strength.

The multi-factor strength models for basalt-fiber-reinforced concrete soil and ordinary concrete are presented in Figure 21 and Figure 22, respectively. Although some fitting constant terms P are statistically insignificant, the R^2^ values of the models are exceptionally high, reaching 0.99, 0.9994, 0.9963, and 0.9994. This suggests that the models demonstrate excellent overall data fitting performance, and the relationships between variables exhibit a certain degree of reliability. In the actual project, through the data coupling of concrete parameters, the construction organization can be better carried out, so that the concrete structure can be more efficiently applied. For example, when analyzing the lining structure of a tunnel project or the stress on a concrete bridge deck, the bearing capacity of the concrete structure can be analyzed according to the coupling mechanism, which provides a reliable basis for construction and ensures its safety.

## 6. Conclusions

This research systematically investigates the coupling mechanism between pore evolution and mechanical strength development in basalt-fiber-reinforced concrete through a novel integration of X-ray computed tomography and the watershed algorithm. By establishing a gray-scale threshold-based pore extraction method, the dynamic interactions between pore structure and strength were quantitatively analyzed across multiple curing ages. The key findings are summarized as follows:

(1) BFRC exhibited superior ductility, characterized by a yielding-like phase before fracture, contrasting the brittle failure of NC. The three-dimensional fiber network effectively bridged micro-cracks, redistributed stress, and enhanced energy dissipation, thereby improving structural resilience.
(2) The incorporation of 0.2% basalt fiber significantly improved the splitting tensile strengths of concrete. At 28 days, BFRC exhibited a compressive strength of 50.78 MPa and splitting tensile strength of 4.07 MPa, surpassing ordinary concrete by 19.88% and 43.3%, respectively. Early strength reduction in BFRC (13.13 MPa vs. 22.81 MPa for NC at 3 days) was attributed to fiber-induced interference during initial hydration, which diminished as hydration progressed, leading to accelerated strength gain post 7 days.
(3) The integration of CT scanning with the watershed algorithm enabled the precise segmentation of pores and the concrete matrix, overcoming the subjectivity of traditional threshold methods. This approach provided critical insights into the development of the pore structure over the hydration age, validated by high-resolution imaging and statistical analysis.
(4) BFRC demonstrated a 64.83% lower porosity (5.13%) compared to NC (11.66%) at 28 days. Microscopic analysis revealed that harmless pores (<1.104 × 10^7^ μm^3^) dominated BFRC, increasing from 42.8% to 77.5%, whereas NC showed a rise from 33.1% to 67.6%. This refinement was driven by the densification of inter-facial transition zones, where basalt fibers promoted the directional growth of hydration products, effectively filling capillary pores and suppressing harmful pore formation.
(5) A two-dimensional strength-porosity model and a three-dimensional age-dependent multi-factor model were developed. The latter achieved exceptional predictive accuracy (R^2^ up to 0.9968) by incorporating the curing time and porosity interactions, offering a robust tool for optimizing the fiber-reinforced concrete design.
(6) Future research directions: (1) Study the hydration process of concrete mixed with different fibers and the effect of different fibers on the hydration process of concrete. (2) Employ experimental methodologies to systematically examine the correlation between mechanical properties and pore structures in recycled aggregate basalt-fiber-reinforced concrete, with the objective of facilitating its industrial implementation in the context of global energy conservation and carbon emission reduction initiatives.

## Figures and Tables

**Table 1:** Mix proportion of concrete (kg/m^3^).

Experimental Group (1 m^3^)	Water	Cement	Sand	Stone	Basalt Fiber
NC	197.8	394.4	632.73	1175.07	0
BFRC	197.8	394.4	632.73	1175.07	0.2%/5.3 kg

**Table 2:** Sample quantity table.

Experimental Group	Compressive Strength Test	Splitting Tensile Test	CT Scan
NC	24	24	3
BFRC	24	24	3

**Table 3:** Compressive strength of specimen.

Type	Compressive Strength (MPa)							
	3 Days	5 Days	7 Days	9 Days	11 Days	14 Days	21 Days	28 Days
BFRC	13.13	29.75	35.45	40.35	42.83	46.2	48.1	50.78
NC	22.81	27.53	31.23	35.68	37.84	39.51	41.53	42.36

**Table 4:** Splitting tensile strength of specimen.

Type	Splitting Tensile Strength (MPa)							
	3 Days	5 Days	7 Days	9 Days	11 Days	14 Days	21 Days	28 Days
BFRC	1.87	2.89	3.47	3.63	3.69	3.80	3.88	4.07
NC	1.53	1.75	1.93	2.21	2.42	2.61	2.72	2.84

## Data Availability

The original contributions presented in this study are included in the article. Further inquiries can be directed to the corresponding author.
